# Novel food resources and conservation of ecological interactions between the Andean Araucaria and the Austral parakeet

**DOI:** 10.1002/ece3.9455

**Published:** 2022-10-27

**Authors:** Guillermo Blanco, Pedro Romero‐Vidal, José L. Tella, Fernando Hiraldo

**Affiliations:** ^1^ Department of Evolutionary Ecology Museo Nacional de Ciencias Naturales (CSIC) Madrid Spain; ^2^ Department of Physical, Chemical and Natural Systems Universidad Pablo de Olavide Sevilla Spain; ^3^ Department of Conservation Biology Estación Biológica de Doñana (CSIC) Sevilla Spain

**Keywords:** *Araucaria araucana*, *Enicognathus ferrugineous*, exotic plants, feeding switch, masting seed crops, novel interactions, Patagonian Andes, urban habitats

## Abstract

In fragile ecosystems, the introduction of exotic species could alter some ecological processes. The Austral parakeet (*Enicognathus ferrugineous*) shows close ecological and evolutionary relationships with the Andean Araucaria (*Araucaria araucana*), so any alteration in these interactions may have negative consequences for both partners and for ecosystem functioning and structure. We conducted extensive roadside surveys to estimate the abundance of parakeets in the northern Patagonian Andes over 4 years and recorded the food plants consumed by foraging flocks. The use of native habitats and humanized areas like villages and farms was influenced by the Araucaria seed crop. In masting years, the large seed crop allowed a massive use of this resource during the non‐breeding season, and even during the breeding season. The exploitation of exotic plants was minor in the masting year, but became predominant in non‐masting years, especially during the non‐breeding season. This feeding switch towards exotic plants primarily arose because the low Araucaria seed crop in non‐masting years is entirely consumed just after production by domestic and wild exotic mammals living in Araucaria forests year‐round, thus forcing the displacement of parakeets towards anthropic habitats to exploit exotic plants. Given the degradation of the remaining Andean Araucaria forests due to the impact of exotic mammals on the ecological interaction between Araucaria and Austral parakeets, ambitious programs to exclude or reduce the density of these alien mammals, including livestock, are warranted.

## Graphical abstract

The results of this study show that the inter‐annual dependence of Austral parakeets on native habitats is influenced by the masting seed production strategy of Araucaria, a key species in the northern Patagonian Andes. Currently, this dependence seems to be largely modulated by disturbing factors promoting a feeding shift to exotic plants in anthropic habitats, especially during non‐masting years.

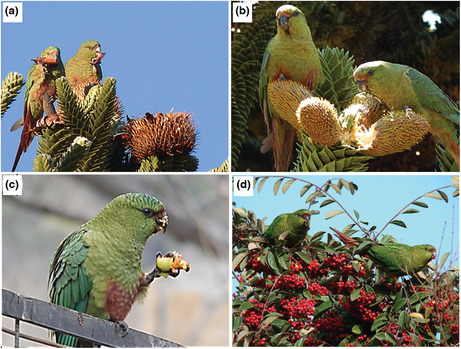

## 1 INTRODUCTION

Land‐use changes and habitat degradation are widespread in most ecosystems (Fahrig, 2003; Maxwell et al., 2016; Sala et al., 2000). Among these impacts, urbanization and farming are increasingly driving biodiversity loss worldwide (Alberti, 2015; Zabel et al., 2019). These large‐scale transformations decrease native plant diversity and abundance, while favoring exotic plants used in agriculture, forestry and urban gardening (David et al., 2017; Davis, 2009; Kennedy et al., 2018; Vitousek et al., 1996). The comprehensive understanding of how wildlife responds to these anthropogenic alterations is a key challenge in ecology and conservation biology. In particular, some wild species can suffer from the combined effects of land‐use change and the introduction of exotic invasive species, which can disrupt ecological and evolutionary interactions in unprecedented ways (Sala et al., 2000). Whereas close ecological interactions might be lost due to the introduction of exotic species (e.g. lack of seed dispersal by native species due to predation by exotic ones), others may arise when these species permeate food chains by providing new resources (Harvey et al., 2010; Pearson & Callaway, 2003; Valentine et al., 2020). Generalist and mobile species can partially cope with habitat changes and periods of native food shortage by consuming exotic resources (Kremen et al., 2007; Muñoz et al., 2007; Páez et al., 2018). Whether this adaptability allows native and invasive species to survive and maintain viable populations depends on multiple and interplaying factors, including ecological factors such as increased risk of predation, reduced availability or quality of food, enhanced competition leading to contest and energetic demands, and anthropogenic factors such as contamination, persecution, collision with infrastructures, and habitat transformation, among others (Robertson et al., 2013; Williams et al., 2017). Thus, a comprehensive evaluation is required for a full understanding of the ecological and conservation implications of novel resources for wildlife.

Among generalist plant consumers, psittacines (Psittaciformes) have been recently highlighted as exerting an important role in ecosystem structure and functioning (Baños‐Villalba et al., 2017; Blanco et al., 2015; Young et al., 2012). Wide spatial–temporal variations in food distribution, availability and nutritional requirements force psittacines to frequently change the composition of food resources (Benavidez et al., 2018; Juniper & Parr, 2010; Renton et al., 2015; Toft & Wright, 2015). These often include exotic plants introduced in multiple ecosystems, for which psittacines can even act as pollinators and seed dispersers (Blanco et al., 2018, 2020; Hernández‐Brito et al., 2021; Young et al., 2012). Psittacines have introduced themselves as exotic species globally (Calzada Preston & Pruett‐Jones, 2021; Mori & Menchetti, 2021), which demonstrates their ability to prosper by feeding on plant species with which they have not evolved (Matuzak et al., 2008; Toft & Wright, 2015). The loss and degradation of native ecosystems have been highlighted as promoting parrot exploitation of cultivated crops, which raises a potential conflict with concerning implications for the conservation of threatened species (Barbosa et al., 2021; Blanco et al., 2021). However, whether the scarcity of particular food resources in native ecosystems promotes a feeding shift to exploit novel resources from exotic species remains poorly understood.

Highly seasonal Southern Andean forests show low plant diversity and offer scarce food resources for plant consumers during Austral winter (Díaz, 2012; Dzendoletas et al., 2003). In this biome, the Austral Monkey puzzle (*Araucaria araucana*) is a keystone species with which the Austral parakeet (*Enicognathus ferrugineous*) has had a close ecological and evolutionary relationship (Gleiser et al., 2017, 2019; Tella, Lambertucci, et al., 2016). The Austral Monkey puzzle (Araucaria in Spanish) shows a masting seed production strategy (Sanguinetti & Kitzberger, 2008). After being shed during autumn, seeds remain on the ground during winter and spring depending on crop abundance, especially due to masting versus non‐masting crop production (Shepherd & Ditgen, 2012; Speziale et al., 2018; Tella, Lambertucci, et al., 2016). By producing temporally‐concentrated high quantities of large seeds during autumn, especially in particular years (masting years), the plant is able to satiate predators then acting as the main seed dispersers (Tella, Lambertucci, et al., 2016). The Austral parakeet pollinates female cones (Gleiser et al., 2017, 2019), and consumes and disperses large quantities of seeds, to the point that it is considered the most important long‐distance disperser of this species (Shepherd & Ditgen, 2012; Speziale et al., 2018; Tella, Lambertucci, et al., 2016). Seed production is highly variable among years, being particularly high during masting when seeds are available during a large part of the year, and thus represent a primary resource for the population of Austral parakeet (Shepherd et al., 2008; Speziale et al., 2018; Tella, Lambertucci, et al., 2016). These seeds are also exploited by a rich variety of exotic, invasive and domestic, mammals ranging in size from rats to large livestock like cattle and equids (Sanguinetti & Kitzberger, 2010; Shepherd & Ditgen, 2005, 2012; Tella, Lambertucci, et al., 2016, see also Dénes et al., 2018 for *Araucaria angustifolia*). These exotic species may consume large quantities of seeds and deplete the crop in years of low production, which imply a threat to the Austral parakeet population largely dependent on this crucial resource during the non‐breeding season (Tella, Lambertucci, et al., 2016).

In this study, we evaluated the variation in the relative abundance and food consumed by Austral parakeets depending on the seed production of Monkey puzzle (hereafter Araucaria), including a masting year. We aimed to explore whether food depletion (Araucaria seeds) due to exotic mammals can induce a feeding shift in the Austral parakeet towards exotic plant species in urban and cultivated areas during the critical non‐breeding season and whether this depends on the production of Araucaria seeds in masting versus non‐masting years. We hypothesized that the natural low diversity of alternative trophic resources during the non‐breeding season, further reduced by native habitat destruction and fragmentation, could force Austral parakeets to search for novel food resources represented by exotic plant species in urban and cultivated areas when Araucaria seeds are not available in non‐masting years due to the consumption by exotic mammals. Therefore, we predict that the dependence on food from urban and other anthropic habitats should be especially high in years of low production of Araucaria seeds (non‐masting years). We discuss whether these novel food resources from exotic species can represent a suitable opportunity, although with potential threats associated with anthropogenic environments, for Austral parakeet populations, and the ecological consequences for seed dispersal.

## 2 MATERIALS AND METHODS

### 2.1 Study area

**FIGURE 1:**
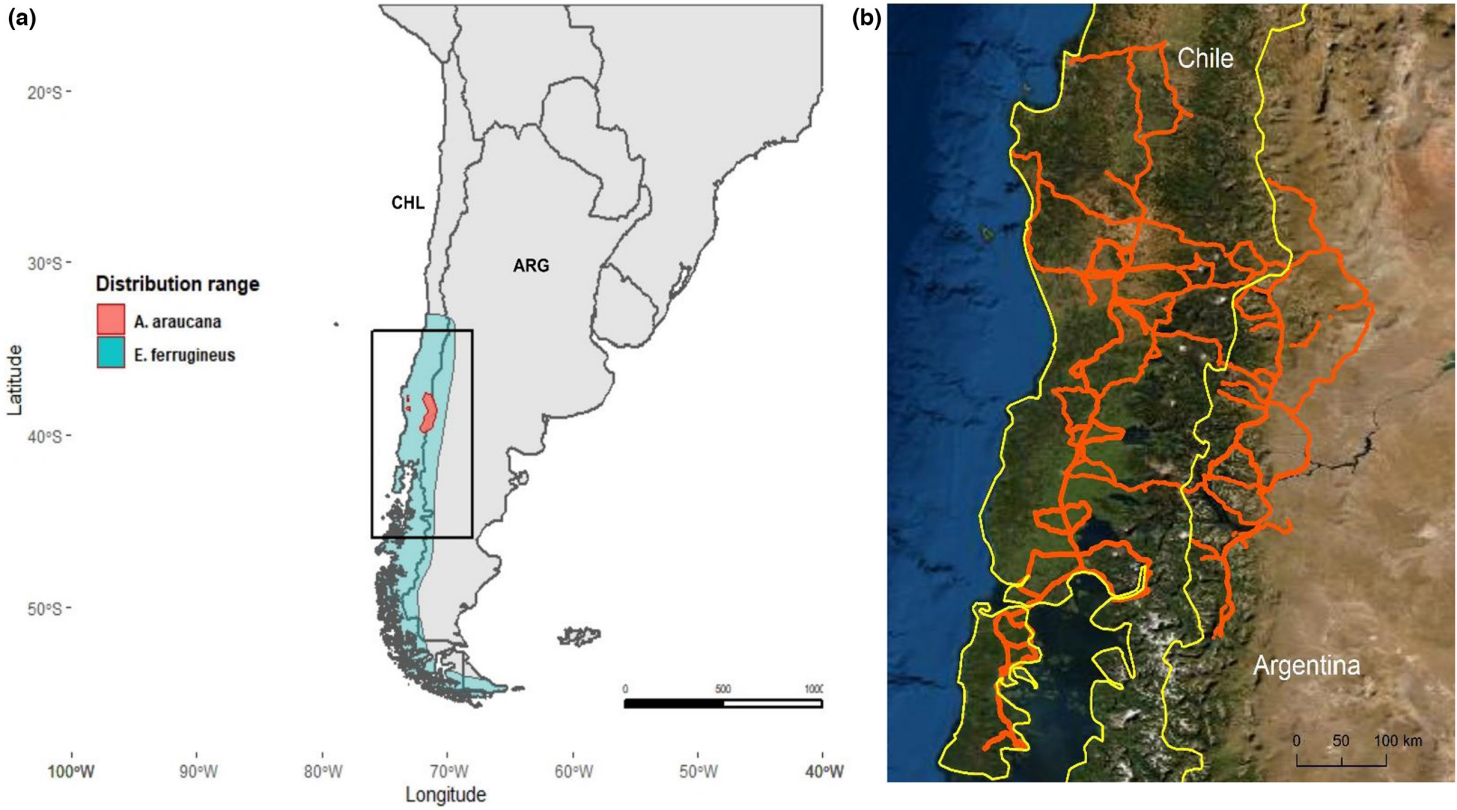
(a) Distribution range of Austral parakeets (blue area) and Araucaria (orange area); (b) Study area showing the roads surveyed (red lines) to estimate the abundance and foraging habits of Austral parakeets in northern Patagonian Andes and lowland surroundings in Argentina and Chile (yellow line represents the border between the two countries).

The study was conducted in north‐western Patagonia, both in Argentina and Chile, covering two Biomes (Temperate Broadleaf & Mixed Forests, and Temperate Grasslands, Savannas & Shrublands) which correspond to two Ecoregions (Valdivian temperate forests and Patagonian steppe, respectively) according to https://ecoregions2017.appspot.com/ (accessed 12 July 2021). Several National Parks are present in the area: Nahuel Huapi and Lanín in Argentina, Huerquehue, Villarica, Conguillo and Nahuelbuta in Chile (Figure 1a,b). The area represents a pronounced environmental gradient with three distinct dominant physiognomic units from west to east: forests, scrublands, and steppes (Figure 1). Forests are particularly represented by Araucaria and *Nothofagus* sp. pure and mixed forests. The area occupied by Araucaria forests has been reduced to only 392 km^2^, mostly in the Valdivian temperate forests ecoregion, due to habitat destruction (Premoli et al., 2013), while agricultural (especially cereals in Chile), forestry (with fast‐growing conifers and eucalypts), livestock production, and urban areas have been growing within and outside of the protected areas during the last decades. The climate presents well‐marked seasons: a warm and dry season from December to February with average temperatures around 17–19°C, a colder season from March to May, and a cold and rainy season from the end of May to September, with average temperatures of 7–8°C (Paruelo et al., 1998). Within and surrounding the national parks protecting native Araucaria and *Nothofagus* forests, a growing human population is altering the landscape by increasing urban areas and introduction of exotic plants used for gardening (Rovere et al., 2013). Exotic plants in urban areas in the study area can cover relatively large and growing areas, due to the extensive pattern of urban development, which is dominated by single‐family houses with gardens, and areas of garden plants along streets. Due to the relatively low diversity of trees and shrubs in the Patagonian steppe, urban nuclei may represent “islands” of exotic plant diversity providing novel foods for Austral parakeets.

### 2.2 Study species

The Araucaria is an endangered conifer tree endemic to the southern Andes (Hoffmann, 1991; Premoli et al., 2013). This dioecious (rarely monoecious) species reaches sexual maturity at 20–30 years old. Females produce cones ranging on average from <1 per tree in non‐masting years to more than 60 per tree in masting years. Cones carry between 100 and 200 seeds each, weighing 3.5 g on average (Sanguinetti & Kitzberger, 2008), but there can be as little as 0.2 cones per tree in non‐masting years producing only 58 seeds (Sanguinetti, 2014). This increase in seed production is a highly synchronized event creating regional mastings across most of the Araucaria distribution when it occurs, but seed production is variable across this distribution (Sanguinetti, 2014). Seed ripening occurs from February to May, peaking in April (Donoso, 2006). The seeds are rich in carbohydrates, particularly starch, and fat (Bergesse et al., 2020; Conforti & Lupano, 2011; Henríquez et al., 2008).

During the study period, seed production in the area varied markedly, with 2013 being a year of high seed production or regional masting, with 62 cones per tree across most of the Araucaria distribution (hereafter masting year). The remaining inter‐mast study years (hereafter non‐masting years) showed low production with 1 and 2.9 cones/tree in 2014 and 2015, respectively (except for a local masting year in 2014 in Pino Hachado and Villa Pehuenia, at the north of the distribution range) or medium production (2016; 41 cones/tree; Sanguinetti, 2014, 2021).

**FIGURE 2:**
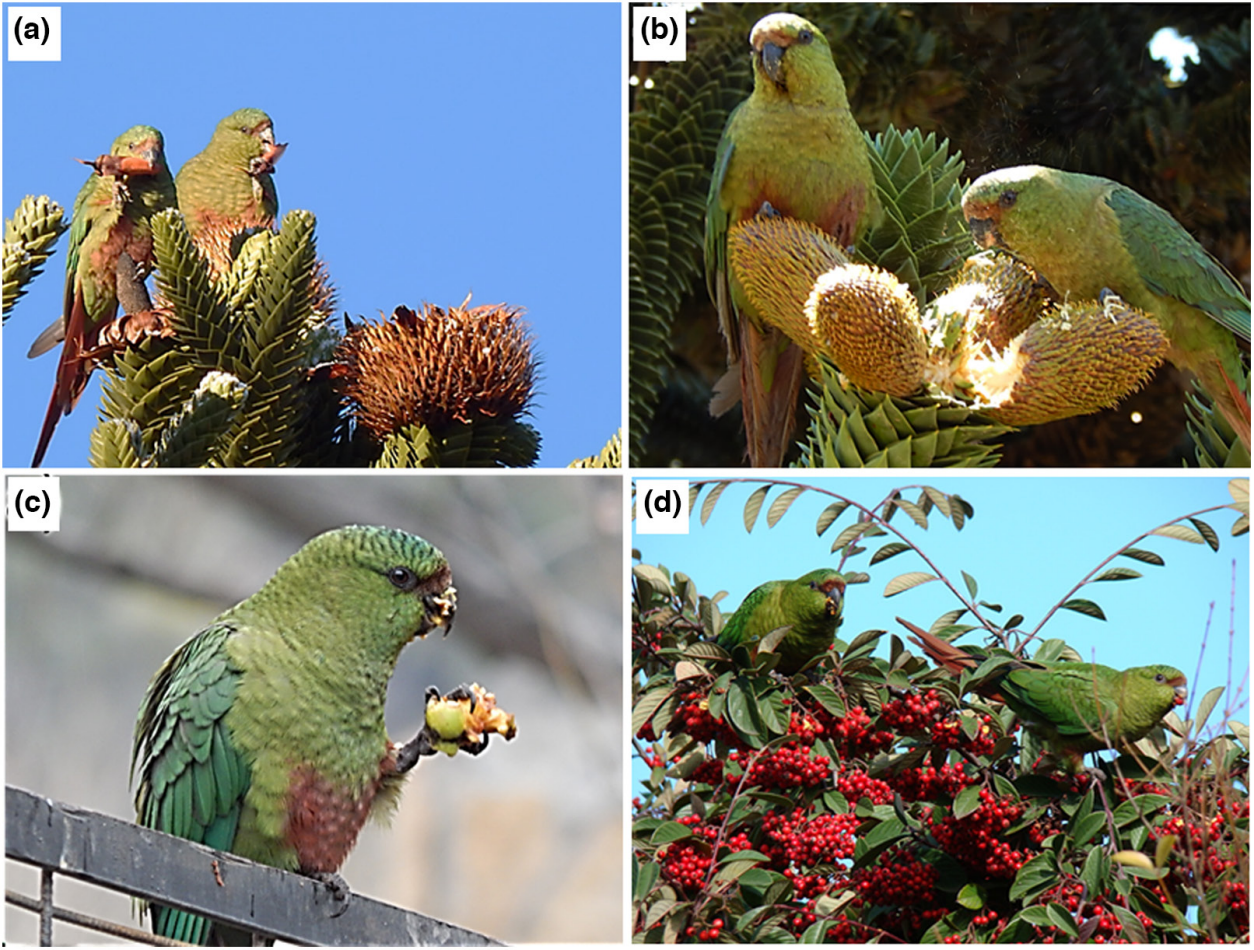
Austral parakeets feeding on (a) Araucaria seeds and (b) pollen from Araucaria male cones in native forests, and fruits of exotic (c) *Malus domestica* and (d) *Cotoneaster* sp.in urban areas of northern Patagonian Andes. Photographs: Orlando Mastrantuoni.

The Austral parakeet (Figure 2) is the only parrot species inhabiting Araucaria forests. Austral parakeets feed on seeds directly taken from Araucaria trees before primary dispersal by barochory, and later on fallen seeds after moving them to distant perches, thus dispersing the seeds (Tella, Lambertucci, et al., 2016). While the primary dispersal rate performed by this species is extremely low (0.1% of the seeds picked out from the tree), secondary dispersal (i.e., after mature seeds fall to the ground) reaches 57% of the picked seeds, with dispersal distances ranging between 5 and 50 m (mean 15 m) (Tella, Lambertucci, et al., 2016). However, dispersal distances were clearly underestimated since only minimum distances up to where the seed‐carrying flying parakeet went out of sight could be recorded (Tella et al., 2019). They also feed on the pollen and sap of Araucaria, as well as on seeds, flowers, pollen, buds and invertebrates of other native species (Díaz et al., 2012; Díaz & Kitzberger, 2006; Díaz & Peris, 2011; Tella, Lambertucci, et al., 2016). The reproductive season starts in the austral middle‐late spring, with laying in late December (Díaz, 2012). Year‐round roadside surveys (Tella et al., 2021) showed a higher relative abundance (individuals/km) in the Valdivian temperate forests (0.75 in Argentina, 1.15 in Chile) than in the Patagonian steppe ecoregion (0.26 in Argentina), likely due to a higher abundance of Araucaria in the former ecoregion (Hoffmann, 1991; Premoli et al., 2013).

### 2.3 Fieldwork

Estimating parrot abundance in the wild is a challenging task, and there are a variety of methods with different pros and cons, the choice of which depends on the research goals of each particular study. We choose roadside surveys since this methodology allows the sampling of large areas to increase the likelihood of recording highly gregarious parrot species showing patchy distributions (Tella et al., 2021), as it is the case of the Austral parakeet. The number of individuals recorded using this method strongly correlates (*r* = .93) with density estimates accounting for differences in detectability (i.e., through distance‐sampling modeling), and allows the calculation of relative abundances (individuals/km) for species that yield insufficient encounters for modeling detectability (see further details, strengths, and weakness of this method in Tella et al., 2021). We drove along unpaved and little transited roads at low speed (20–40 km/h), avoiding the central hours of the day (from about 12:00 to 15:00 h depending on the season) and bad weather, to count Austral parakeets and recording their feeding behavior, following the methodology used in previous studies (Tella et al., 2021). The censuses were carried out during the breeding season of Austral parakeets (November–February) and the non‐breeding season (March–October) from 2013 to 2016. Overall, roadside surveys were conducted on 12,884 km during 110 fieldwork days by two to three persons in eight expeditions (four in the breeding season and four in the non‐breeding season) covering most of the study area (Figure 1b). Surveys over the same roads were sometimes conducted, but always in different years and seasons to avoid double counting, and they represent a very small part of the distance surveyed. Furthermore, this does not mean that the same surveys were repeated, as it depended on the sampling schedule and environmental conditions at the time they were run.

During roadside surveys, we recorded the beginning and the end of each patch of different habitats and its length (in km) (Carrete et al., 2009; Tella et al., 2021). These patches were categorized into five major categories: two human‐modified habitats (agricultural/farming; urban), and three native ones (pure forest of *Nothofagus* spp.; pure and mixed native forest with Araucaria – Araucaria hereafter – and steppe‐scrubland). This field‐based habitat classification represents a general approximation of the actual fine‐grained composition of the landscape due to its heterogeneous nature, often including a variable mixture of habitat patches changing over years as a consequence of the continuous degradation and transformation of natural habitats by human activities. To deal with this challenge of habitat classification, we relied on the dominant habitat in each patch and divided the surveys into as many patches as necessary depending on the often frequent changes in habitat composition, which was assessed in situ based on visual observation on both sides of the road traveled. We used these five habitat categories for graphical representation of raw data. For statistical modeling (see below), we created the variable *habitat type* with two levels: anthropic (pooling the two anthropogenic habitats) and native (pooling the three native environments). Abundance by habitat was estimated as the total number of parakeets recorded divided by the kilometers surveyed, considering each habitat patch as the sampling unit (Tella et al., 2021).

When Austral parakeets were detected feeding, both while conducting roadside surveys and when moving between roadside survey areas, we stopped to record the following data: number of individuals (hereafter flock size, including single foraging individuals), plant species, item exploited in the case of Araucaria, and habitat. Stops were extended long enough (generally 5–10 min) to record this information by coordinating the activities of the two or three observers, following the methods described previously (Tella, Lambertucci, et al., 2016). When needed after the observations were resolved, we approached the target plants on which the parakeets were feeding to obtain additional information (traits of the items consumed, the identity of the plant species, photographs and measurements of fruits, seeds, flowers, and leaves for identification confirmation). In this form, we attempted to confirm what parakeets were eating and wasting by using the necessary time to search for food remains on the ground beneath foraging sites (Sebastian‐Gonzalez et al., 2019), photographing the remains and the mother plant for later identification using guides to the flora of the region and exotic species (Bisheimer, 2012; Bisheimer & Fernández, 2009; Sanz & Valente, 2005; Zuloaga & Belgrano, 2016). Foraging observations were conducted within and outside surveys conducted to determine the abundance of Austral parakeets. Plant species were classified as native or exotic according to Zuloaga and Belgrano (2016). Foraging habitat was registered in the five categories used in the surveys of abundance (see above). Overall, we recorded 354 foraging flocks, summing c. 20,000 individuals, and the plant species and other matter the flocks were consuming, including foraging flocks recorded during the abundance surveys and during additional observations outside surveys.

During the breeding and non‐breeding season of the non‐masting years (2014–2016), the seed abundance below the same marked trees (*n* = 516) sampled in the masting year (2013) was recorded by using the same methodology (Tella, Lambertucci, et al., 2016). To evaluate the specific food resources exploited by Araucaria, we distinguished the consumption of seeds from other food items, pooling pollen from male cones, sap, leaves and bark. Because it was not possible to determine exactly what resources each individual of each flock was feeding, we recorded the food item most commonly exploited by each flock after the snapshot observation of most flock members, which was later confirmed by searching for food remains beneath the exploited Araucaria trees. For several flocks, it was not possible to determine the part of the Araucaria on which the parakeets were mostly feeding; therefore, the sample size (number of flocks) slightly differed between analyses.

### 2.4 Data analysis

We used generalized linear models (GLM) (negative binomial error distribution; logit link function) to analyze the abundance of parakeets according to season (categorized in breeding and non‐breeding seasons), habitat, Araucaria seed production (categorized in masting and non‐masting years), and their interactions. Given the scarcity of parakeet observations in some combinations of habitat × season × Araucaria crop production categories, we simplified the statistical analysis by pooling habitats into native or anthropic (see above). We used as a response variable the number of parakeets recorded in each habitat patch across surveys, by using the 2225 habitat patches surveyed as the sample unit. Therefore, we included patch length (in km) as a covariate to control for survey effort in each patch. The ecoregion (Patagonian steppe or Valdivian temperate forests) in which each transect was included was fitted in models as a fixed factor, given the expected differences in the relative abundance of parakeets between them (see above). The analysis was run using the package glmmTMB in R to correct for zero‐inflation (Brooks et al., 2017), given that parakeets were not recorded in most (89%) of the 2225 habitat patches surveyed.

The frequency of use of each habitat (pooling anthropic [agricultural and urban] vs. native habitats) by flocks of foraging parakeets (*n* = 354) was analyzed according to season and Araucaria seed production by the mean of Fisher's exact test. Factors affecting the exploitation of native versus exotic plants were analyzed by GLM using the binomial error distribution (native = 0, exotic = 1) and the logistic link function, by considering the flock (*n* = 354) as the analysis unit. Season, ecoregion, and the abundance of Araucaria seed production (masting or non‐masting years) were considered as predictor variables (fixed factors).

To explore the specific importance of Araucaria as a key resource for Austral parakeets depending on season and masting seed production, we conducted a log linear analysis to examine the relationship between more than two categorical variables. We grouped food species into three categories (Araucaria, other native species, and exotic species), while two categories were used for season (breeding and non‐breeding) and Araucaria seed production (masting and non‐masting years). The analysis was conducted in a hierarchical fashion, starting with the three‐order interaction, and then proceeding backward until all two‐order interactions maintained in the model reached statistical significance (*p* < .05) according to the *χ* ^2^ likelihood ratio.

Foraging flock size variation was assessed with a GLM (truncated negative binomial error distribution, logit link function) where the season, ecoregion, Araucaria seed production (masting or non‐masting years), and type of food (native or exotic) were considered as explanatory factors.

Statistical analyses and checking of model assumptions were performed using SPSS software v. 26 (IBM SPSS Statistics) for Fisher's exact test and log‐linear analysis, and the R statistical platform (R Core Team, 2019) for GLMs. Model selection was performed using the Akaike Information Criterion corrected for small sample sizes (AICc; Sugiura, 1978). In each subset of competing models derived after testing each possible combination of covariates (including the null model but not those models that did not converge), the ΔAICc was computed as the difference between the AICc of each model and that of the best model, and the Akaike weight (w) of each model using the AICcmodavg package (Mazerolle, 2020). The resulting models with ΔAICc < 2 were considered as equally supported, and thus were averaged by means of a model averaging procedure using the MuMIn package (Barton, 2020). The fit of the models was evaluated using the package DHARMa (Hartig, 2018).

## 3 RESULTS

### 3.1 Abundance of parakeets

**FIGURE 3:**
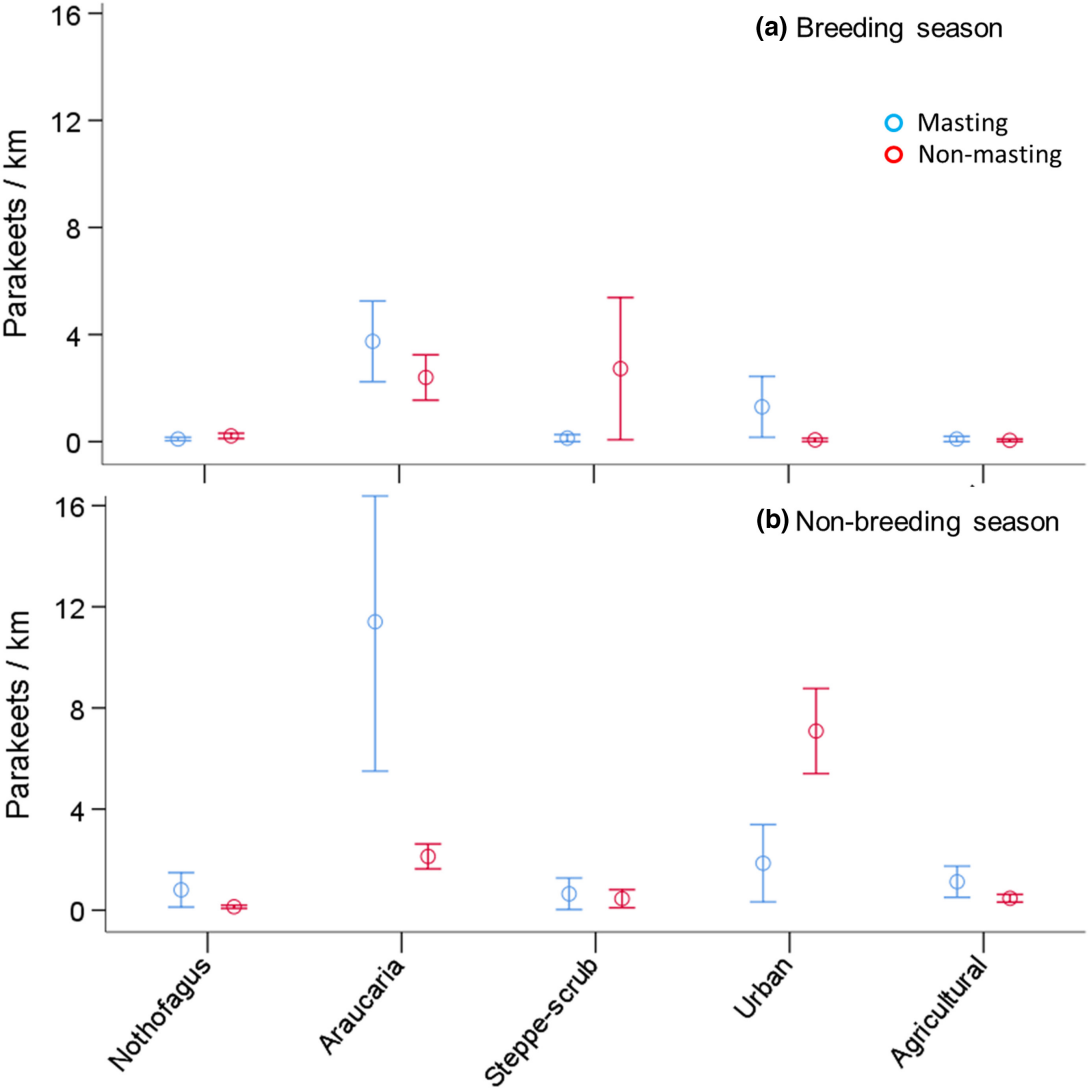
Relative abundance of Austral parakeet (mean ± SE individuals per km) recorded during roadside car surveys in each habitat type according to Araucaria crop abundance (masting or non‐masting) during the (a) breeding season and (b) non‐breeding season in northern Patagonian Andes. See Table 1 for the statistical results explaining the main effects.

Overall, we recorded 11,808 Austral parakeets during roadside surveys across 12,884 km, covering 2225 habitat patches across 4 years, including foraging, perching and flying flocks. Parakeets were patchily concentrated (they were only recorded in 11% of the patches surveyed) and not evenly distributed across habitats, seasons and years. The averaged conditional count model obtained (Table 1) showed that parakeet relative abundance was lower in the breeding season (Figure 3a) than in the non‐breeding seasons (Figure 3b), differed between habitats depending on Araucaria seed production (interaction between habitat and masting – non‐masting years) while controlling for patch length and ecoregion. This effect was especially patent in the non‐breeding season due to the higher relative abundance in Araucaria forests and urban areas in masting and non‐masting years respectively (Figure 3b). The averaged conditional zero‐inflated model obtained (Table 1), which models zero values (i.e., patches with no parakeets recorded) indicated a lower occupancy of patches in the masting year and in the Patagonian steppe ecoregion while controlling for patch length.

**TABLE 1:** Results from the zero‐inflated negative binomial GLM fitted to explain variability in the abundance of Austral parakeets after averaging the best‐supported models.

Predictors	Estimates	SE	Incidence rate ratios	CI	*p*
Conditional count model					
(Intercept)	2.82	0.27	16.74	9.87–28.40	<.001
Patch size (km)	0.01	0.01	1.01	0.99–1.02	.217
Araucaria: masting	0.11	0.18	1.11	0.77–1.60	.564
Season: non‐breeding	0.49	0.21	1.64	1.08–2.49	.020
Habitat: native	−0.01	0.12	0.99	0.78–1.25	.928
Ecoregion: Patagonian steppe	−0.32	0.23	0.72	0.46–1.14	.162
Araucaria (masting) × Habitat (native)	0.36	0.14	1.43	1.09–1.87	.010
Season (non‐breeding) × Habitat (native)	0.30	0.22	1.34	0.87–2.07	.180
Season (non‐breeding) × Araucaria (masting)	0.03	0.12	1.03	0.82–1.29	.805
Conditional zero‐inflated model					
(Intercept)	2.16	0.21	8.70	5.81–13.03	<.001
Patch size (km)	−0.02	0.01	0.98	0.97–0.99	<.001
Araucaria: masting	0.32	0.09	1.38	1.17–1.64	<.001
Ecoregion: Patagonian steppe	0.30	0.19	1.34	0.92–1.96	.125

*Note*: See Tables S1 and S2 for model selection and Figures S1 and S2 for model fits.

### 3.2 Foraging habitats and feeding patterns

**FIGURE 4:**
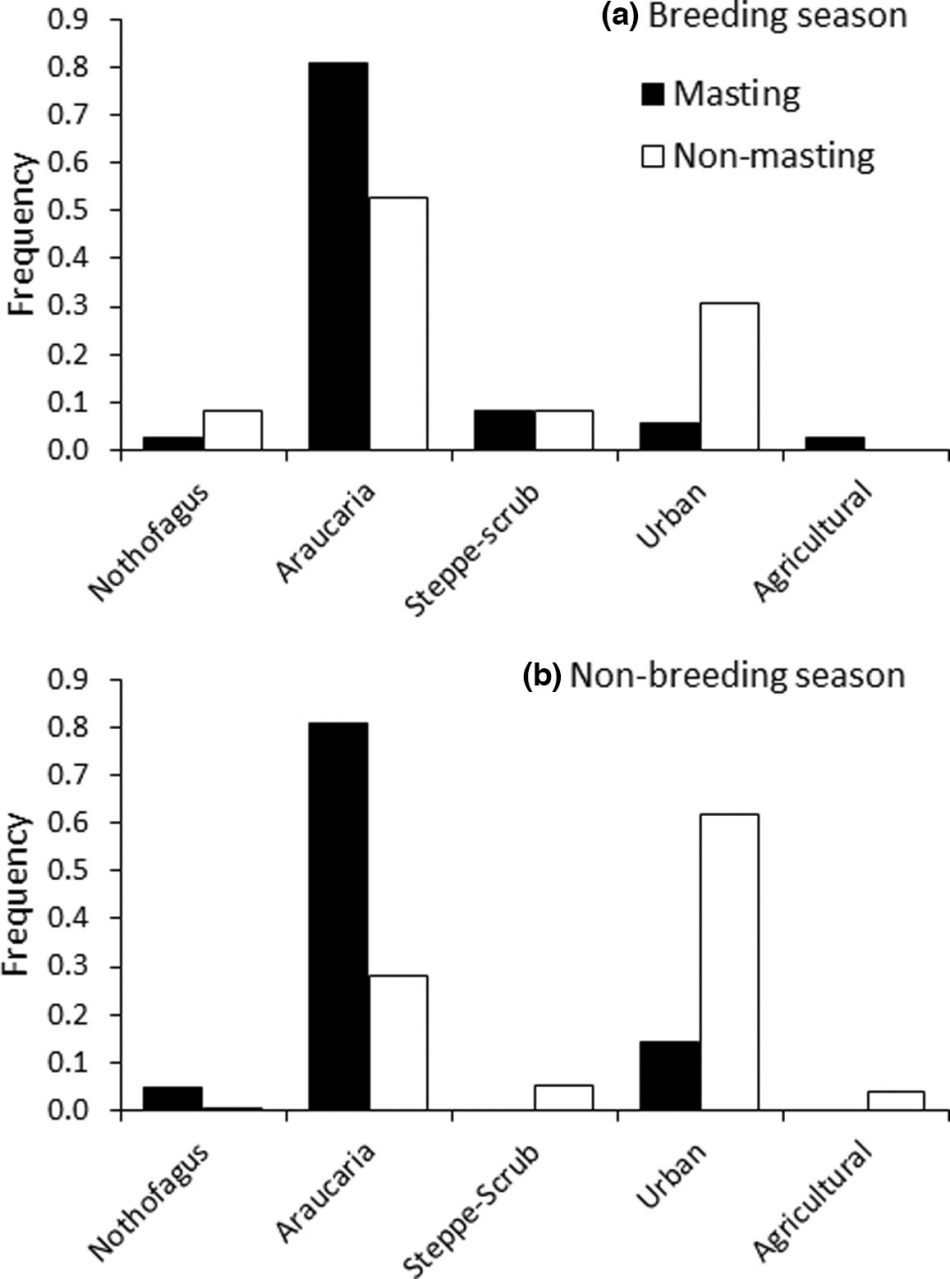
Frequency of Austral parakeet flocks (*n* = 354, including single individuals) foraging in each habitat type according to Araucaria crop abundance (masting or non‐masting) during the (a) breeding season and (b) non‐breeding season in northern Patagonian Andes.

Austral parakeets fed in all habitat types considered. A high proportion of flocks exploited native, pure and mixed Araucaria and *Nothofagus* forests during the breeding season, especially in the masting year, while the frequency of use of urban areas increased in the non‐masting years (Figure 4a). Pooling anthropic (agricultural and urban) versus native habitats, the frequency of use of native habitats was higher in the masting year (91.9%, *n* = 37) than in the non‐masting years (69.4%, *n* = 36) during the breeding season (Fisher's exact test, *p* = .019). In the non‐breeding season, the same pattern of habitat use was found (Figure 4b) but the difference in the use of anthropic and native habitats in masting (85.7% of flocks in native habitats, *n* = 21) or non‐masting years (34.2%, *n* = 260) was more acute (Fisher's exact test, *p* < .0001).

Overall, we recorded 354 foraging flocks, summing c. 20,000 individuals, and the plant species and other matter the flocks were consuming, including foraging flocks recorded during the abundance surveys and during additional observations outside surveys. We recorded Austral parakeets feeding on at least 18 identified native and 21 exotic plant species (several identified to the genus and family level), including angiosperms and gymnosperms (Table 2). Among native species, Araucaria, *Nothofagus* spp., and *Acaena splendens* (Table 2) were the most exploited. Among exotic species, *Populus* spp., *Prunus* spp. and *Malus domestica* were the plants most exploited (Table 2). Parakeets consumed a variety of plant food items, including Araucaria seeds (Figure 2a), pollen (Figure 2b) and sap, as well as seeds, fruits, flowers, flower buds, leaves, leaf buds, sap, bark and twigs of native and exotic plants, including wild herbs and cultivated cereal grain, fruit trees (Figure 2c) and garden plants (Figure 2d) in urban areas. They also fed on *Nothofagus* hemiparasites (*Misodendrum* spp.), fungi (*Cyttaria* spp.), lichens (*Usnea* spp.) and invertebrates (Table 2). We also observed parakeets ingesting pebbles likely used as gastrolits.

**TABLE 2:** Diet composition of the Austral parakeet in breeding and non‐breeding areas in years of masting and non‐masting seed crops of Araucaria, according to flocks (F) and individuals (I).

Food species	Masting		Non‐masting		Total, F/I
	Breeding, F/I	Non‐breeding, F/I	Breeding, F/I	Non‐breeding, F/I	
Native species					
*Araucaria araucana*	19/431	13/960	9/117	40/1550	81/3058
*Nothofagus* spp.		4/129	3/48	24/1959	31/2136
*Acaena splendens*	8/419		2/40	3/72	13/531
Invertebrates	3/56	1/1	4/81		8/138
*Berberis* spp.	1/25			5/128	6/153
*Cyttaria* spp.	1/1	1/37	1/24	2/230	5/292
*Poaceae* spp.			1/12	4/210	5/222
*Misodendrum* spp.	1/10		4/69		5/79
*Lomatia hirsute*	1/10			3/97	4/107
*Embothrium coccineum*	1/12		2/58	1/23	4/93
*Raphitamnus* sp.				3/78	3/78
*Prumnopitys andina*				2/270	2/270
*Maytenus boaria*			1/8	17/20	2/28
*Gevuina avellana*				1/70	1/70
*Luma apiculate*				1/70	1/70
*Drimys winteri*				1/60	1/60
*Gaultheria phillyreifolia*				1/60	1/60
*Usnea* spp.				1/60	1/60
*Sarmienta scandens*				1/55	1/55
*Ovidia andina*				1/23	1/23
*Laureliopsis philippiana*				1/14	1/14
Exotic species					
*Populus* spp.		2/18		62/3044	64/3062
*Prunus* spp	1/1		3/17	22/2570	26/2588
*Malus domestica*				19/1397	19/1397
*Castanea sativa*				12/1164	12/1164
*Pinus ponderosa*			2/24	8/260	10/284
Cereal grain			1/12	8/549	9/561
*Taraxacum officinale*	1/2		3/53	4/380	8/435
*Juglans regia*				6/826	6/826
*Quercus robur*				5/492	5/492
*Betula pendula*				3/460	3/460
*Rosa rubiginosa*				3/52	3/52
*Fraxinus* spp.				2/188	2/188
*Acer pseudoplatanus*				2/14	2/14
*Crataegus monogyna*				1/300	1/300
*Pyracantha* sp.				1/300	1/300
*Ficus carica*				1/120	1/120
*Cotoneaster* sp.				1/100	1/100
*Liquidambar* sp.				1/100	1/100
*Pyrus domestica*				1/20	1/20
*Sambucus nigra*				1/20	1/20
*Ilex* sp.				1/1	1/1
Total	37/967	21/1145	36/563	260/17,406	354/20,081

About half of the foraging flocks (*n* = 172, summing 7375 individuals) were feeding on native species while the other half of flocks (*n* = 182 flocks, summing 12,706 individuals) were fed on exotic species (Table 2). According to the averaged GLM obtained (Table 3), the exploitation of exotic plants by flocks (*n* = 354) was lower in masting (93.1% native, 6.9% exotic, *n* = 58 flocks) than in non‐masting years (39.5% native, 60.5% exotic, *n* = 296 flocks), while controlling for a higher consumption, although not statistically significant, of exotic plants in the Patagonian steppe ecoregion and during the non‐breeding season (Table 3). Although the model included the interaction of season × Araucaria seed production, the interaction was not significant (Table 3).

**TABLE 3:** Results from the binomial GLM fitted to explain the consumption of exotic plants by Austral parakeets after averaging the two best‐supported models.

Predictors	Estimates	SE	Odds ratio	CI	*p*
(Intercept)	−0.50	0.61	0.60	0.18–2.00	.409
Ecoregion: Patagonian steppe	0.89	0.55	2.45	0.83–7.17	.103
Season: breeding	−0.67	0.24	0.51	0.32–0.82	.005
Araucaria: masting	−1.20	0.28	0.30	0.17–0.52	<.001
Season (breeding) × Araucaria (masting)	0.24	0.28	1.27	0.74–2.20	.386

*Note*: See Table S3 for model selection and Figure S3 for model fits.

**FIGURE 5:**
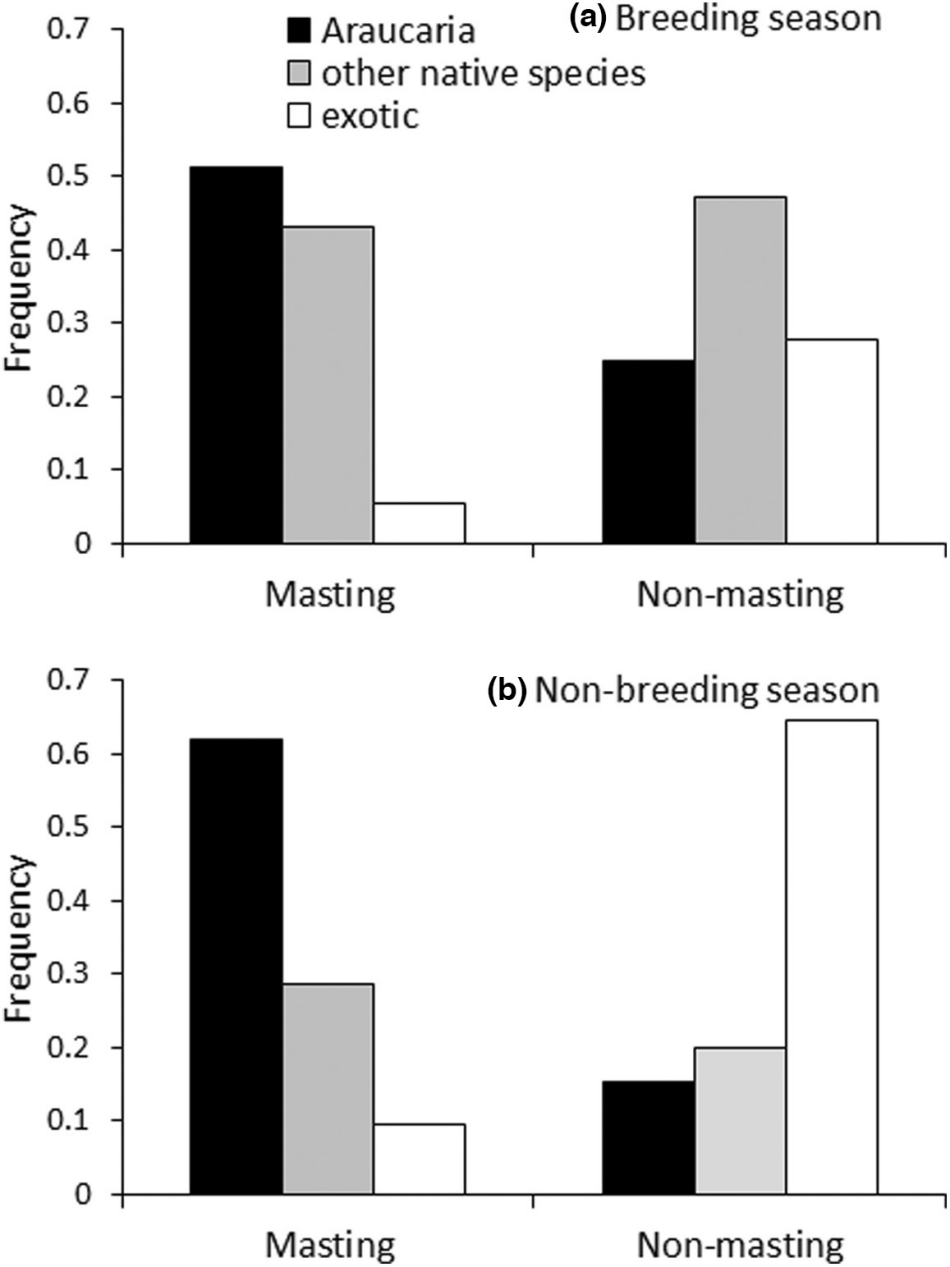
Frequency of Austral parakeet flocks (*n* = 354, including singe individuals) foraging on Araucaria, other native plant species, and exotic species according to Araucaria crop abundance (masting or non‐masting) during the (a) breeding season and (b) non‐breeding season in northern Patagonian Andes. Significant interactions between food type and Araucaria seed production, and between food type and season, were found according to a log‐linear analysis (see Section 3.3).

A log‐linear analysis taking into account simultaneously the relationships between food type (Araucaria, other native species, and exotic species), season and Araucaria seed production showed no significant three‐way interactions (*χ* ^2^ = 0.65, df = 2, *p* = .72). Significant interactions between food type and Araucaria seed production (*χ* ^2^ = 38.79, df = 2, *p* < .001), and between food type and season (*χ* ^2^ = 19.05, df = 2, *p* < .001) were found. The fit of the model was adequate (*χ* ^2^ = 0.65, df = 2, *p* = .72). These results show that in the breeding season Austral parakeets mostly foraged on native species, especially on Araucaria during the masting year (Figure 5a). In the non‐breeding season, Araucaria was exploited predominantly in masting years, while exotic plants were exploited mostly in non‐masting years (Figure 5b).

### 3.3 Foraging flock sizes

**FIGURE 6:**
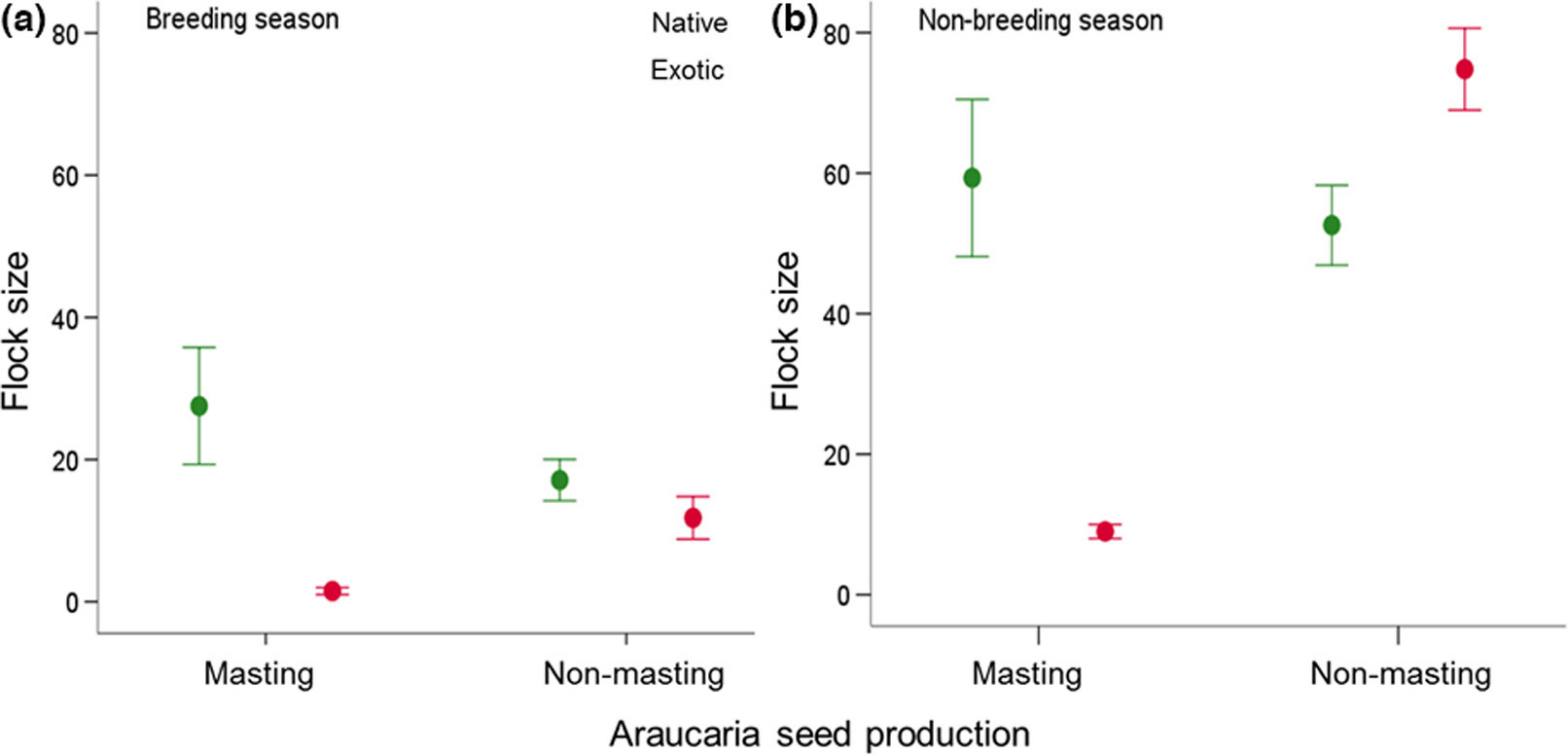
Flock size (mean ± SE) of Austral parakeets (*n* = 354, including singe individuals) feeding on native and exotic plant species according to Araucaria seed production (masting or non‐masting) during the (a) breeding season and (b) non‐breeding season in northern Patagonian Andes. See Table 4 for the statistical results explaining the main effects.

Individual Austral parakeets forage as single individuals or flocks of up to 300 individuals (mean ± SD number of foraging individuals in each observation event = 56.7 ± 65.4, median = 30, *n* = 354). According to the averaged GLM model, flock size was influenced by food type (native or exotic), Araucaria seed production (masting or non‐masting) and their interaction, and season, while controlling for the smaller flock sizes in the Patagonian steppe ecoregion (Table 4). This indicates that flock size was smaller in the breeding season (Figure 6a) than in the non‐breeding season (Figure 6b). During the breeding season, flocks exploiting native species were larger in masting than non‐masting years (Figure 6a). In addition, flocks were larger when parakeets exploited native plants (especially Araucaria) in the non‐breeding season of the masting year than in the non‐masting years (Figure 6b), and when they exploited exotic plants in the non‐breeding season of non‐masting years (Figure 6b).

**TABLE 4:** Results from the truncated negative binomial GLM fitted to explain variability in foraging flock size of Austral parakeets after averaging the two best‐supported models.

Predictors	Estimate	SE	Incidence rate ratio	CI	*p*
(Intercept)	2.47	0.24	11.95	7.4–19.30	<.001
Ecoregion: Patagonian steppe	−0.46	0.19	0.63	0.44–0.91	.014
Season: breeding	−0.59	0.09	0.54	0.45–0.65	<.001
Food: native	0.58	0.16	1.81	1.32–2.48	<.001
Araucaria: masting	−0.55	0.16	0.57	0.41–0.78	<.001
Food (native) × Araucaria (masting)	0.75	0.16	2.16	1.58–2.95	<.001
Season (breeding) × Araucaria (masting)	0.05	0.09	1.13	0.94–1.36	.531

*Note*: See Table S4 for model selection and Figure S4 for model fits.

### 3.4 Exploitation of food items from Araucaria

When exploiting food resources provided by Araucaria, Austral parakeets fed exclusively on seeds during the non‐breeding season, both in masting (100% of flocks, *n* = 9, summing 730 individuals) and non‐masting years (100% of flocks, *n* = 37, 1262 individuals). In contrast, they fed on both seeds and other food resources (pollen, sap and leaves) during the breeding season of the masting year (26.3% on seeds, *n* = 5 flocks, 186 individuals, and 73.7% on other food resources, *n* = 14 flocks, 400 individuals, respectively), but exclusively on other food resources in non‐masting years (100%, *n* = 7 flocks, 90 individuals) due to the lack of available seeds on the ground, as assessed by searching below a large sample of marked and randomly selected trees. Overall, the consumption of seeds and other food differed between masting and non‐masting years (Fisher exact test, *p* < .0001).

## 4 DISCUSSION

The results of this study show that the inter‐annual dependence of Austral parakeets on native habitats is influenced by the masting seed production strategy of Araucaria, a key species in the northern Patagonian Andes. Currently, this dependence seems to be largely modulated by anthropogenic impacts on native habitats promoting a feeding shift to exotic plants in anthropic habitats, especially during non‐masting years. According to the typical seasonal and habitat‐associated abundances of psittacines linked to the tracking of food resources, the Austral parakeet diet varied among habitats, seasons and years with and without Araucaria masting seed crops, showing a much wider feeding breath than previously reported (Díaz & Kitzberger, 2006; Díaz & Peris, 2011). Food resources included a high variety of native and exotic species in response to land use changes (Barbosa et al., 2021; Salinas‐Melgoza et al., 2013). These included most growth forms of plants and their resources located from overground to canopy in each habitat. They also exploited hemiparasite mistletoes, tree fungi, lichens and unidentified invertebrates. Therefore, as with other psittacine species, Austral parakeets behave as trophic generalists providing a variety of ecological functions (Blanco et al., 2018; Toft & Wright, 2015) that are expected to influence the demography of their food plants, and thus the structure and functioning of ecosystems (Baños‐Villalba et al., 2017; Blanco et al., 2015; Montesinos‐Navarro et al., 2017; Tella et al., 2020).

Austral parakeets foraged in both native habitats and anthropic habitats areas like villages and farms. While the exploitation of exotic plants in anthropic habitats was residual in the masting year, it became predominant in non‐masting years showing a very low abundance of Araucaria seeds (Sanguinetti, 2014), especially during the non‐breeding season, although seeds can be locally available in some patches with medium crops in inter‐masting years. These patterns were also reflected in flock size as a proxy of the coordinated and increased use of concentrated and predictable food resources by psittacines, both wild and cultivated and native or exotic (Barbosa et al., 2021). In fact, flock size was largest when Austral parakeets exploited Araucaria in the non‐breeding season of the masting year, and when exploited exotic plants in the non‐breeding season of non‐masting years. The nomadic and migratory strategies for the exploitation of resources in the highly seasonal environment of the Patagonian Andes could allow Austral parakeets to switch to novel resources exploited in anthropic habitats depending on the availability of native resources, especially Araucaria seeds, and they also exhibit altitudinal movements in response to the availability of resources (Díaz & Kitzberger, 2006). Although movement patterns of this species are not well understood, the high seasonality of Austral environments makes Araucaria crops especially valuable during the harsh conditions of Austral winter, when migratory movements of the parakeets could occur from their southernmost distribution range, up to about 1500 km away (Díaz, 2012; Juniper & Parr, 2010). Research is needed to determine the magnitude of seasonal and inter‐annual migratory and nomadic movements of Austral parakeets depending on food availability in Araucaria forests and other habitats.

Currently, Araucaria forests have lost around 40% of their original distribution area due to overexploitation of wood and pastures (Premoli et al., 2013). However, historically, the wider distribution of the Araucaria forest together with the lack of additional anthropic threats might be able to support the entire population of Austral parakeets during the non‐breeding season. The high climate seasonality and the inter‐annual availability of food resources, especially the Araucaria seed crop, can represent natural key factors regulating the Austral parakeet population, likely by increasing mortality rates during the non‐breeding season of non‐masting years. This could be compensated for by high reproductive rates after masting years. In fact, the number of eggs laid by this species (6.5 eggs in average; Díaz, 2012), which is higher than that laid by other psittacine species of similar size (Juniper & Parr, 2010), could be an adaptation to highly seasonal environments governing food resources and demography of this species.

While switching foraging habitats can help psittacines to cope with fluctuating resource availability in nature (Renton et al., 2015; Toft & Wright, 2015), the exploitation and eventual depletion of a key food resource by domestic and wild exotic species can force the use of novel habitats and resources by native species (Valentine et al., 2020). Our results support these types of novel multi‐faceted interactions derived from human action disrupting ecological and evolutionary relationships, with concerning effects on whole populations and ecosystems. In addition, the Austral parakeet shows close ecological and evolutionary relationships with Araucaria (Gleiser et al., 2017; Tella, Dénes, et al., 2016; Tella, Lambertucci, et al., 2016), which suggests that any alteration of these relationships may have negative consequences for both interacting species with implications for ecosystem functioning and structure (Montesinos‐Navarro et al., 2017). In particular, the massive exploitation and eventual depletion of the Araucaria seed crop by introduced mammals, especially during non‐masting years, limits to masting years the seed dispersal function provided by Austral parakeets, which can have consequences on forest regeneration. This requires further research for a comprehensive understanding. This tight dependence on a particular plant species during a relatively long period of the year, and its functional consequences for the food plant, have been recorded for other psittacines. For instance, the whole population of the Red‐spectacled amazon (*Amazona pretrei*) gathers each year in a very local area to feed on and disperse the seeds of the other Araucaria species in America, the Parana pine (*A. angustifolia*), also exploited and dispersed by other parrot species (Tella, Dénes, et al., 2016). In contrast, the Australian Araucaria with large seeds (Bunya pine, *Araucaria bidwillii*) is mostly dispersed by the Sulfur‐crested cockatoo (*Cacatua galerita*), although this food‐ and habitat‐generalist species only depends on Araucaria seeds locally and during a reduced time period (Tella et al., 2019). Additional close exclusive and asymmetrical interactions between psittacines and keystone plant species include macaws (genus *Ara* and *Anodorhynchus*, and the Red‐bellied macaw, *Orthopsittaca manilatus*) and palms (Arecaceae) with large fruits (Carrete et al., 2022).

We highlight the erosion of pervasive ecological interactions due to human‐induced habitat loss and alteration by exotic invasive species, rather than due to direct human persecution for crop protection and for the pet trade (Barbosa et al., 2021; Romero‐Vidal et al., 2020). The Austral parakeet has been not persecuted in the past by low‐density Araucanian Mapuche and other indigenous people lacking cultivated resources. These human communities lack a cultural tradition of maintenance of parakeets as pets (Rozzi, 2010), probably due to the low ability of Austral parakeets to imitate human speech (see Romero‐Vidal et al., 2020). The recent use of anthropogenic food resources in urban areas implies new challenges and threats inherent to urban life (Alberti, 2015) for the global population of the Austral parakeet, including the conflict with human interests owing to the parakeet's use of fruit trees. Although the actual impact on these crops can be considered low according to our observations (see also Barbosa et al., 2021), the human‐perceived impact of parakeets associated with their habit to forage in large noisy flocks can result in mortality by direct persecution in cultivated and urbanized areas (Díaz, 2012). These habitats are a source of other threats, including contamination with phytosanitary products and other pollutants involved in the intended and unintended poisoning of parakeets, and other mortality risks like collision with windows and cars, electrocution, and predation by domestic cats (Alberti, 2015).

Contrary to the valuable abundant nutrient contents in the form of starch provided by Araucaria seeds (Henríquez et al., 2008; Sanguinetti & Kitzberger, 2008), many of the exotic plants exploited provided fleshy pulp with nutrients that may be especially valuable for maintenance (e.g. sugar carbohydrates) rather than for tissue reserve replenishing, which is essential for winter survival and subsequent migration and reproduction (Karasov & Martínez del Río, 2007; Nuevo, 2015). In addition, Araucaria seeds are larger than those of most other exploited native plant species, except those of *Gevuina avellana*, which is generally distributed in small patches and as isolated individuals. This makes Araucaria seeds an especially valuable resource to fulfill the daily nutritional requirement of parakeets with minimal time spent handling a relatively low number of food items, often partially consumed through a satiation process promoting seed dispersal (Tella, Lambertucci, et al., 2016) and faster germination (Speziale et al., 2018). Therefore, exotic plants exploited in urban areas may not be consumed as a preferred food due to their essential nutritional value, but rather used as an alternative resource for survival when native resources are scarce, especially Araucaria seeds in non‐masting years. If this hypothesis can be proven true, the exploitation of anthropogenic resources from anthropic habitats could represent an ecological trap for the entire population of Austral parakeets. Further research is encouraged to assess the role of Austral parakeets as seed dispersers and pollinators on Andean austral forest structure and functioning, including these ecological functions in Araucaria and other plant species (e.g. Bravo et al., 2020).

## 5 CONCLUSION

Our study highlights the role of introduced species as both consumers (exotic mammals) of novel food resources for them (Araucaria seeds) and providers of novel resources for native species (exotic plants for Austral parakeets). The feeding switch towards exotic plants arose because the low Araucaria seed crop in non‐masting years is entirely consumed just after production by domestic and wild exotic mammals living in Araucaria forests year‐round, thus forcing the displacement of parakeets towards anthropic habitats to exploit exotic plants. Effective methods to reverse the degradation of the remaining Andean Araucaria forests are warranted, including ambitious programs to eradicate invasive alien mammals and to exclude or reduce the density of livestock in this singular and highly threatened ecosystem. The population trend of the Austral parakeet is not known in detail, and there is no accurate information on its long‐distance movements. These questions should be addressed in specific studies monitoring abundance, for which this study can serve as a reference given the large spatial and temporal sampling effort covering years with and without Araucaria masting. Despite the risks inherent in their exploitation, anthropogenic resources could represent a valuable food source in particular seasons or circumstances that could help sustain this and other species. Proper management based on specific research on the actual value of novel resources could help to achieve a positive outcome in terms of conservation.

## AUTHOR CONTRIBUTIONS

**Guillermo Blanco:** Conceptualization (equal); formal analysis (equal); investigation (equal); methodology (equal); supervision (equal); validation (equal); visualization (equal); writing – original draft (equal). **Pedro Romero‐Vidal:** Conceptualization (equal); formal analysis (equal); investigation (equal); methodology (equal); validation (equal); visualization (equal); writing – review and editing (equal). **José L. Tella:** Funding acquisition (equal); investigation (equal); methodology (equal); project administration (equal); supervision (equal); validation (equal); visualization (equal); writing – review and editing (equal). **Fernando Hiraldo:** Conceptualization (equal); data curation (equal); investigation (equal); methodology (equal); supervision (equal); validation (equal); visualization (equal); writing – review and editing (equal).

## CONFLICT OF INTEREST

All authors declare no competing interests.

## Supporting information


Supporting Information

## Data Availability

Relevant data files are archived in the Dryad Digital Repository: https://doi.org/10.5061/dryad.h70rxwdnb.
