# Supplementary material for: Novel food resources and conservation of ecological interactions between the Andean Araucaria and the Austral parakeet
Source: Ecol Evol. 2022 Oct 27;12(10):e9455. doi: 10.1002/ece3.9455 (PMC9608793; doi:10.1002/ece3.9455)
Supplement: Supplementary file 1 — Supporting Information [file ECE3-12-e9455-s001.docx]

**SUPPLEMENTARY MATERIAL**

**Novel food resources and conservation of ecological interactions between the Andean Araucaria and the Austral parakeet**

Guillermo Blanco^1^, Pedro Romero-Vidal^2*^, José L. Tella^3^, Fernando Hiraldo^3^

^1^ Department of Evolutionary Ecology, Museo Nacional de Ciencias Naturales (CSIC),

José Gutiérrez Abascal 2, 28006 Madrid, Spain.

^2^ Department of Physical, Chemical and Natural Systems, Universidad Pablo de Olavide, Carretera de Utrera, km 1, 41013 Sevilla, Spain

^3^ Department of Conservation Biology, Estación Biológica de Doñana (CSIC), Sevilla, Spain.

Corresponding Author:

Pedro Romero-Vidal. Department of Physical, Chemical and Natural Systems, Universidad Pablo de Olavide, Carretera de Utrera, km 1, 41013 Sevilla, Spain

Email: pedroromerovidal123@gmail.com

**1. Model selection**

We selected the most parsimonious models following the Akaike Information Criterion. According to that criterion, models with ∆AICc <2 were averaged by means of a model averaging procedure. Analyses were performed using the function AICcmodavg (Mazerolle 2020) and MuMIn package in R (Barton 2020).

**Table S1**. AIC-based model selection for the zero inflated model to assess sources of variability in the abundance of Austral parakeets. Only models with informative variables are included. The three best-supported models used for model averaging are in bold. Number of estimated parameters (df), log likelihood ratios (logLik), AICc values, AICc differences (∆AICc) compared to the highest ranked model (i.e., the one with the lowest AICc), and Akaike weights (AICcWt) are shown. Explanatory variables: patch size: km; Araucaria: Araucaria seed production (masting or non-masting years); season: breeding or non-breeding season; ecoregion: Valdivian temperate forests or Patagonian steppe.

| Models | df | logLik | AICc | ∆AICc | AICcWt |
| --- | --- | --- | --- | --- | --- |
| Patch size + Araucaria + ecoregion | 13 | -1894.652 | 3815.5 | 0 | 0.259 |
| Patch size + Araucaria + season + ecoregion | 14 | -1893.785 | 3815.8 | 0.29 | 0.224 |
| Patch size + Araucaria + season + habitat + habitat*Araucaria + habitat*season + ecoregion | 17 | -1891.504 | 3817.3 | 1.82 | 0.104 |
| Patch size + Araucaria + habitat + ecoregion | 14 | -1894.651 | 3817.5 | 2.02 | 0.094 |
| Patch size + Araucaria + season + habitat + habitat*Araucaria + ecoregion | 15 | -1893.784 | 3817.8 | 2.32 | 0.081 |
| Patch size + Araucaria + season + habitat + habitat*season + ecoregion | 16 | -1892.833 | 3817.9 | 2.44 | 0.076 |
| Patch size + Araucaria + season + habitat + habitat*Araucaria + season*Araucaria + ecoregion | 17 | -1892.008 | 3818.3 | 2.82 | 0.063 |
| Patch size + Araucaria + season + habitat + habitat*Araucaria + habitat*season + season*Araucaria + ecoregion | 18 | -1891.482 | 3819.3 | 3.81 | 0.039 |
| Patch size + Araucaria + season + habitat + season*Araucaria + ecoregion | 16 | -1893.783 | 3819.8 | 4.34 | 0.03 |
| Patch size + Araucaria + season + habitat + habitat*season + season*Araucaria + ecoregion | 17 | -1892.831 | 3819.9 | 4.47 | 0.028 |
| Patch size + ecoregion | 12 | -1901.357 | 3826.9 | 11.39 | 0.001 |
| Patch size + season + ecoregion | 13 | -1901.304 | 3828.8 | 13.3 | 0 |
| Patch size + habitat + ecoregion | 13 | -1901.357 | 3828.9 | 13.41 | 0 |
| Patch size + season + habitat + ecoregion | 14 | -1901.304 | 3830.8 | 15.33 | 0 |

**Table S2**. AIC-based model selection for the conditional model to assess sources of variability in the abundance of Austral parakeets. Only models with informative variables are included. The four best-supported models used for model averaging are in bold. Number of estimated parameters (df), log likelihood ratios (logLik), AICc values, AICc differences (∆AICc) compared to the highest ranked model (i.e., the one with the lowest AICc), and Akaike weights (AICcWt) are shown. See S1 for the description of explanatory variables.

| Models | df | logLik | AICc | ∆AICc | AICcWt |
| --- | --- | --- | --- | --- | --- |
| Patch size + Araucaria + season + habitat + habitat*Araucaria + ecoregion | 12 | -1894.977 | 3814.1 | 0 | 0.33 |
| Patch size + Araucaria + season + habitat + habitat*season + ecoregion | 12 | -1895.659 | 3815.5 | 1.36 | 0.167 |
| Patch size + Araucaria + season + habitat + habitat*season + habitat*season + ecoregion | 13 | -1894.652 | 3815.5 | 1.37 | 0.166 |
| Patch size + season + season + habitat + habitat*season + Araucaria*season + ecoregion | 13 | -1894.946 | 3816.1 | 1.96 | 0.124 |
| Patch size + Araucaria + season + habitat + season*Araucaria + habitat*season + ecoregion | 13 | -1895.148 | 3816.5 | 2.37 | 0.101 |
| Patch size + Araucaria + season + habitat + habitat*Araucaria + habitat*season + season*Araucaria + ecoregion | 14 | -1894.345 | 3816.9 | 2.79 | 0.082 |
| Patch size + habitat + season + ecoregion | 10 | -1899.96 | 3820 | 5.92 | 0.017 |
| Patch size + Araucaria + season + habitat + ecoregion | 11 | -1899.955 | 3822 | 7.94 | 0.006 |
| Patch size + season + ecoregion | 9 | -1902.824 | 3823.7 | 9.64 | 0.003 |
| Patch size + Araucaria + season + habitat + Araucaria*season + ecoregion | 12 | -1899.835 | 3823.8 | 9.72 | 0.003 |
| Patch size + Araucaria + season + ecoregion | 10 | -1902.6 | 3825.3 | 11.2 | 0.001 |
| Patch size + Araucaria + ecoregion | 9 | -1913.901 | 3845.9 | 31.79 | 0 |
| Patch size + habitat + ecoregion | 9 | -1917.954 | 3854 | 39.89 | 0 |
| Patch size + Araucaria + habitat + ecoregion | 10 | -1917.953 | 3856 | 41.91 | 0 |
| Patch size + ecoregion | 8 | -1920.686 | 3857.4 | 43.34 | 0 |

**Table S3.** AIC-based model selection to assess the effects of season, Araucaria seed production and ecoregion on the consumption of exotic plants by Austral parakeets. Only models with informative variables are included. The two best-supported models used for model averaging are in bold Model selected is represented in bold. Number of estimated parameters (df), log likelihood ratios (logLik), AICc values, AICc differences (∆AICc) compared to the highest ranked model (i.e., the one with the lowest AICc) and Akaike weights (AICcWt) are shown. See S1 for the description of explanatory variables.

| Models | df | logLik | AICc | ∆AICc | AICcWt |
| --- | --- | --- | --- | --- | --- |
| Season + Araucaria + ecoregion | 4 | -202.997 | 414.1 | 0 | 0.659 |
| Season + Araucaria + season*Araucaria + ecoregion | 5 | -202.625 | 415.4 | 1.32 | 0.341 |
| Araucaria + ecoregion | 3 | -211.706 | 429.5 | 15.37 | 0 |
| Season + ecoregion | 3 | -218.469 | 443 | 28.9 | 0 |
| Ecoregion | 2 | -242.262 | 488.6 | 74.45 | 0 |

**Table S4**. AIC-based model selection to assess sources of variability in foraging flock sizes of Austral parakeets. Only models with informative variables are included. The two best-supported models used for model averaging are in bold. Number of estimated parameters (df), log likelihood ratios (logLik), AICc values, AICc differences (∆AICc) compared to the highest ranked model (i.e., the one with the lowest AICc) and Akaike weights (AICcWt) are represented. Food: native or exotic plant; see S1 for the description of other explanatory variables.

| Models | df | logLik | AICc | ∆AICc | AICcWt |
| --- | --- | --- | --- | --- | --- |
| Ecoregion + season + food + Araucaria masting year + Araucaria masting year*food | 7 | -1734.852 | 3484 | 0 | 0.551 |
| Ecoregion + season + Araucaria + food + season*Araucaria + Araucaria*food | 8 | -1734.022 | 3484.5 | 0.43 | 0.444 |
| Ecoregion + season + food | 5 | -1742.588 | 3495.3 | 11.32 | 0.002 |
| Ecoregion + season + Araucaria + season*Araucaria | 6 | -1741.693 | 3495.6 | 11.6 | 0.002 |
| Ecoregion + season | 4 | -1744.188 | 3496.5 | 12.46 | 0.001 |
| Ecoregion + season + Araucaria | 5 | -1743.818 | 3497.8 | 13.78 | 0.001 |
| Ecoregion + food | 4 | -1760.191 | 3528.5 | 44.47 | 0 |
| Ecoregion + Araucaria | 4 | -1764.312 | 3536.7 | 52.71 | 0 |
| Ecoregion | 3 | -1768.771 | 3543.6 | 59.58 | 0 |

**2. Model fits**

Diagnostic tools provided by the DHARMa package in R (Hartig 2021) were used to evaluate the fit of models with ∆AICc <2. DHARMa simulates quantile residuals from a fitted GLM or GLMM that are standardized to values between 0 and 1. For a correctly specified model, these residuals should have a uniform distribution regardless of the underlying model structure and can be interpreted similarly as residuals for linear models. The package includes statistical tests on the residuals to check for uniformity, overdispersion, and zero inflation.

**Figure S1**. qq-plot and standard residuals plots for models with ∆AICc <2 included on the conditional average model to describe the relationship between parrot abundance and the season, Araucaria seed production, habitat, patch size, and ecoregion. No significant problems were detected.


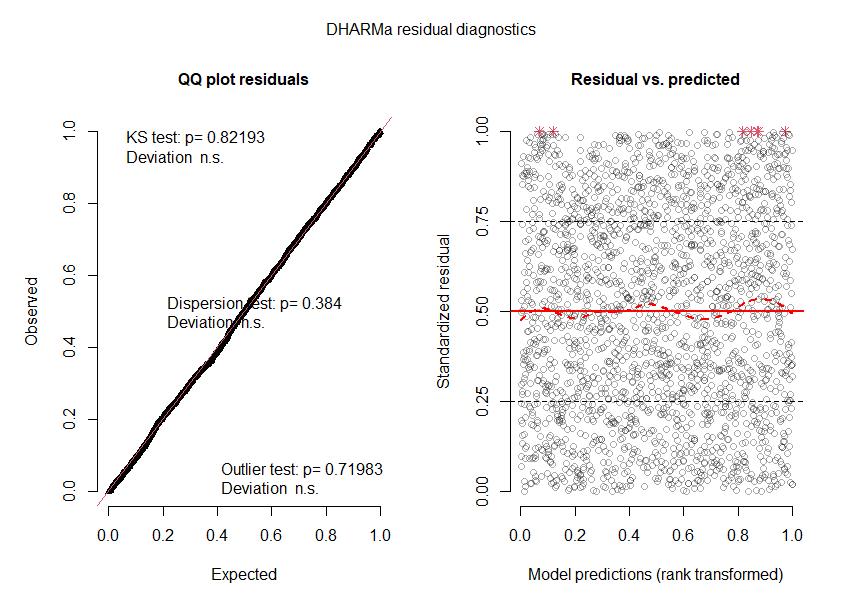


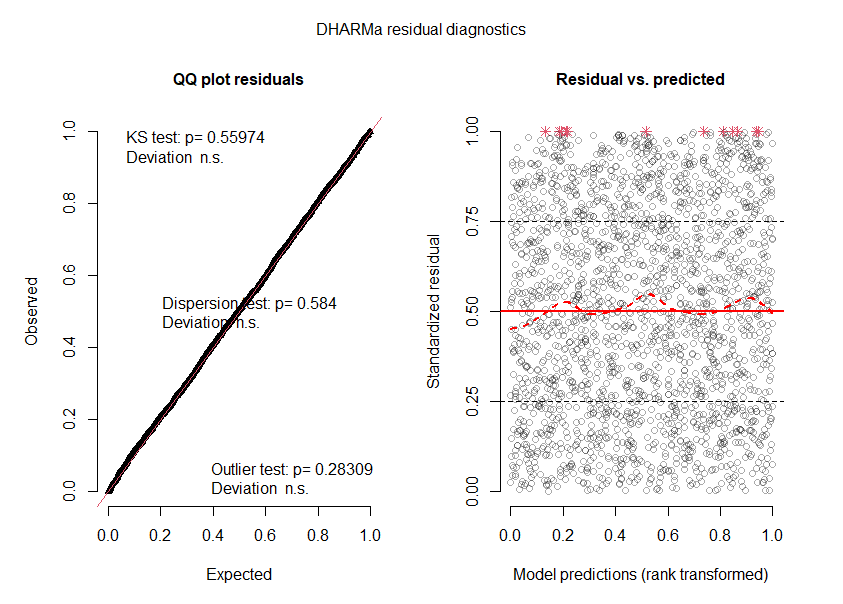


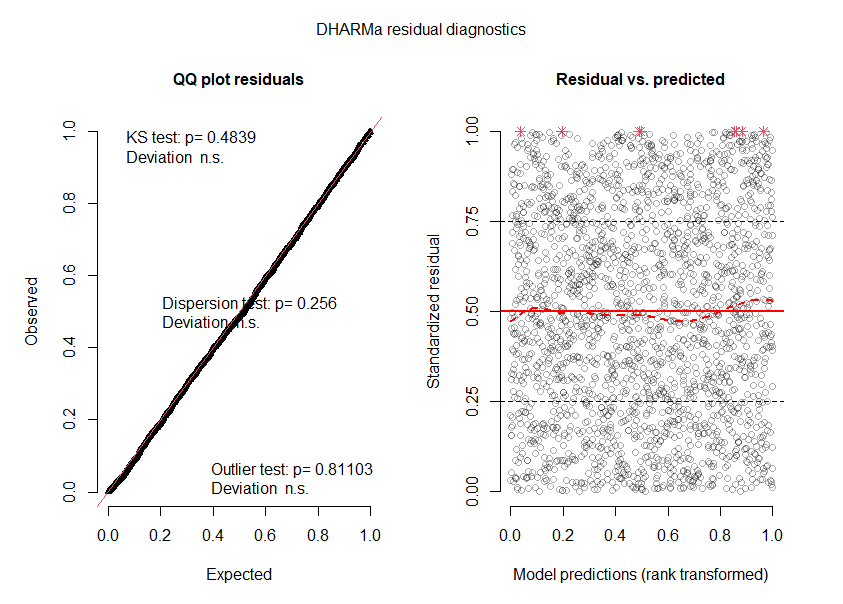


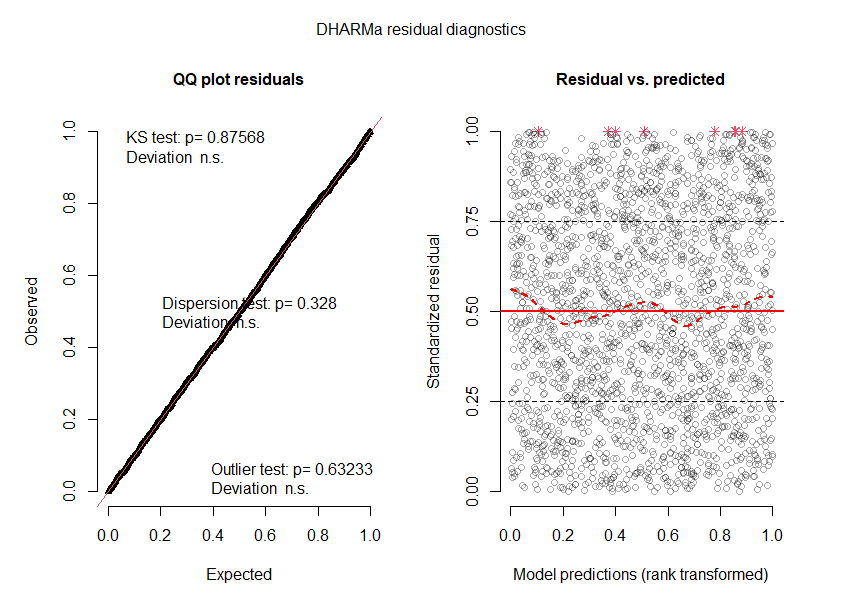


**Figure S2.** Within group deviation from uniformity test for Araucaria, habitat, season and ecoregion for models with ∆AICc <2 included on the conditional average model to describe their relationships with the abundance of Austral parakeets. No significant problems were detected.


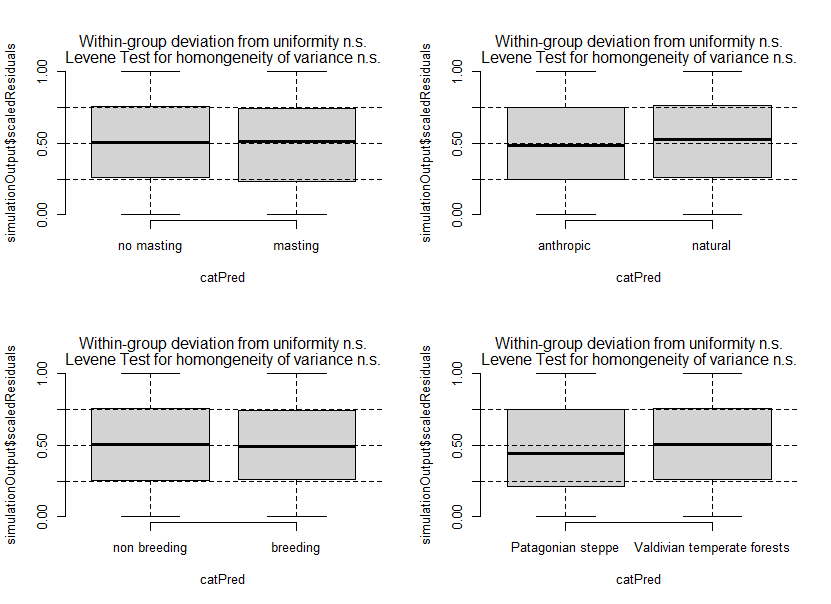


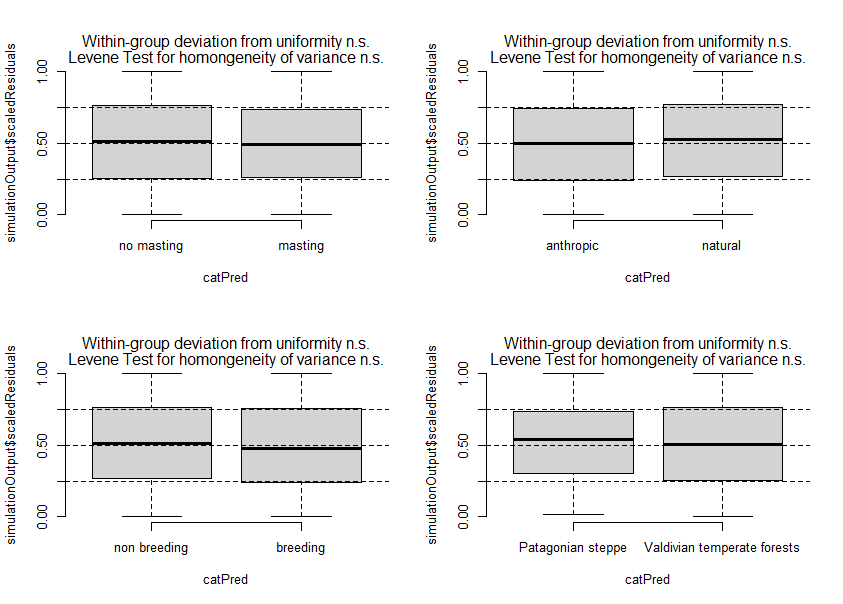


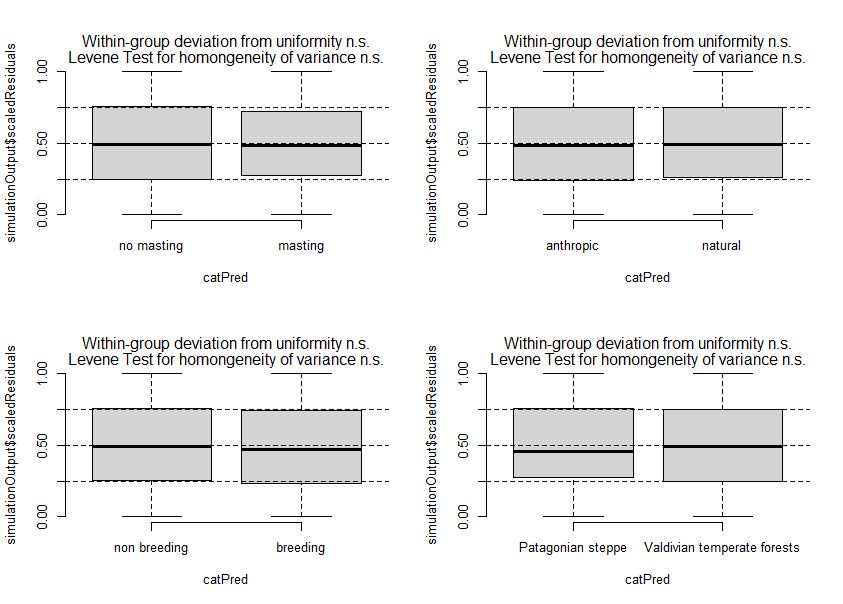


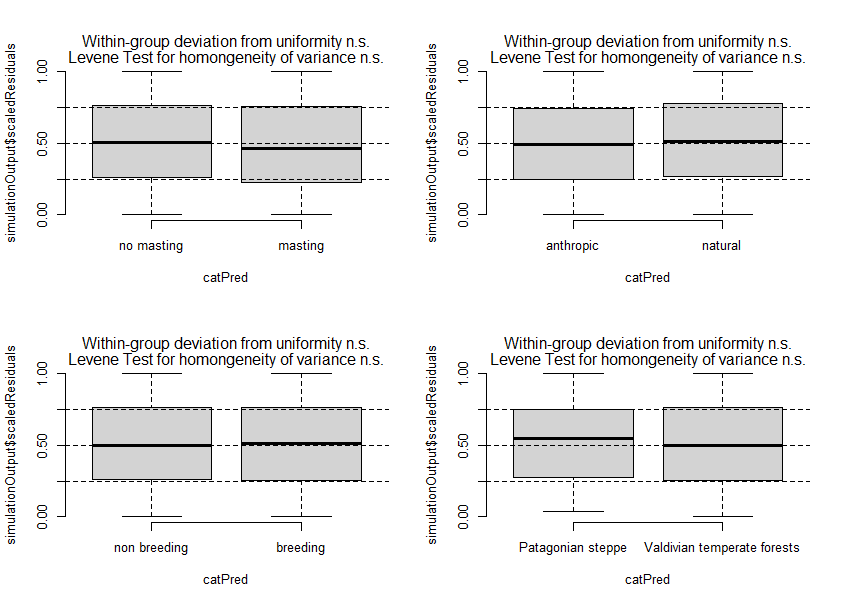


**Figure S3**. qq-plot and Within group deviation from uniformity test for models with ∆AICc <2 included on the conditional average model to describe the relationship between the consumption of native or exotic plants by Austral parakeets and season, Araucaria seed production, and ecoregion. No significant problems were detected.


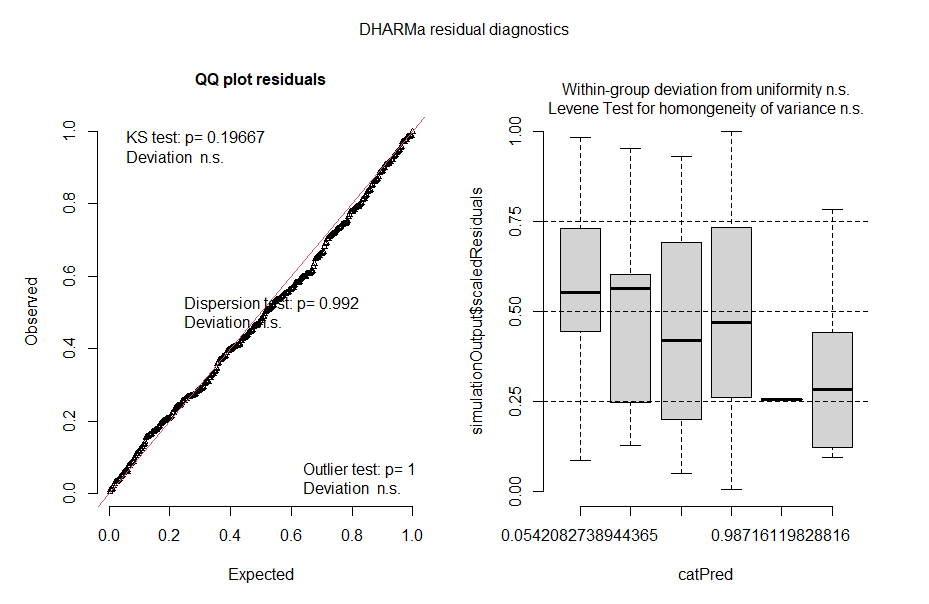


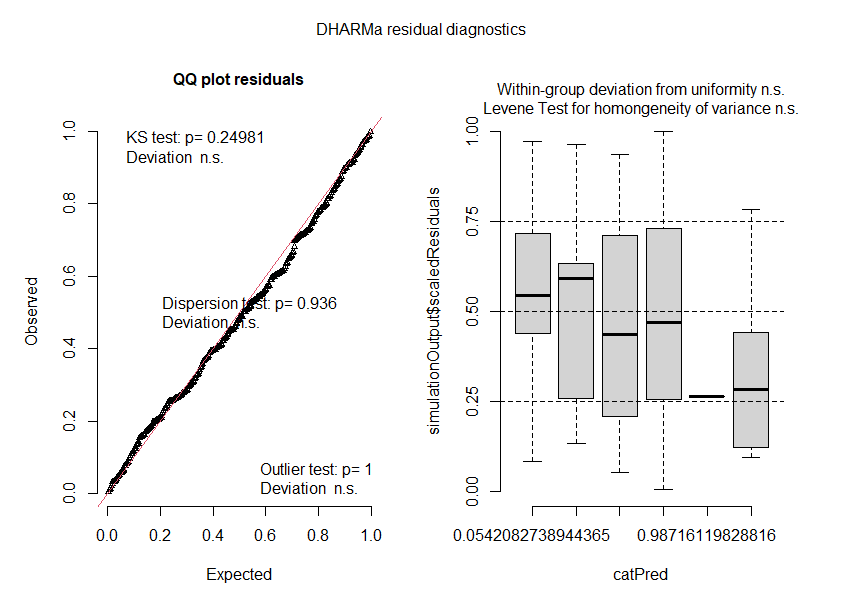


**Figure S4**. qq-plot and standard residuals plots for models with ∆AICc <2 included on the conditional average model to describe the relationship between foraging flock size and season, Araucaria seed production, origin of plants (native or exotic), and ecoregion. No significant problems were detected.


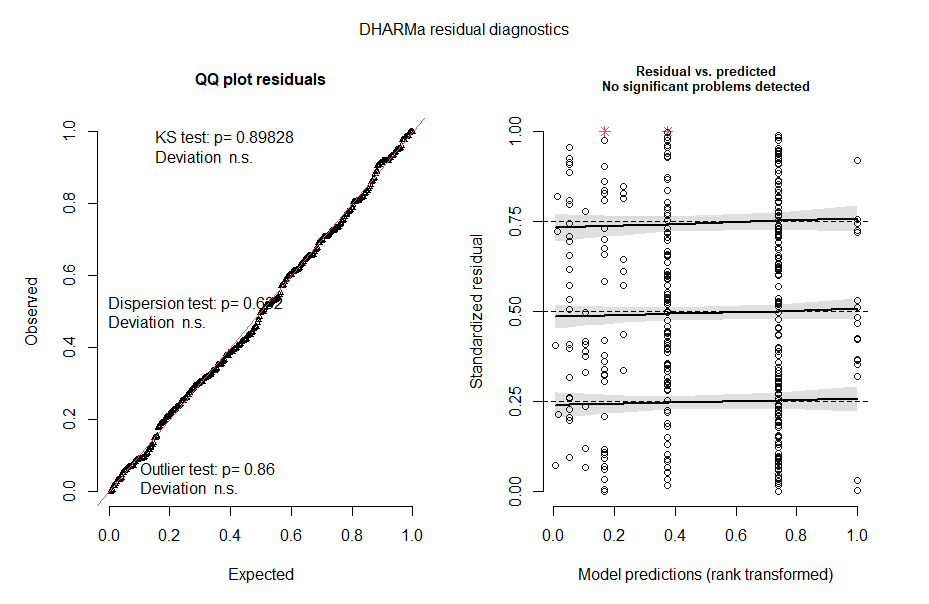


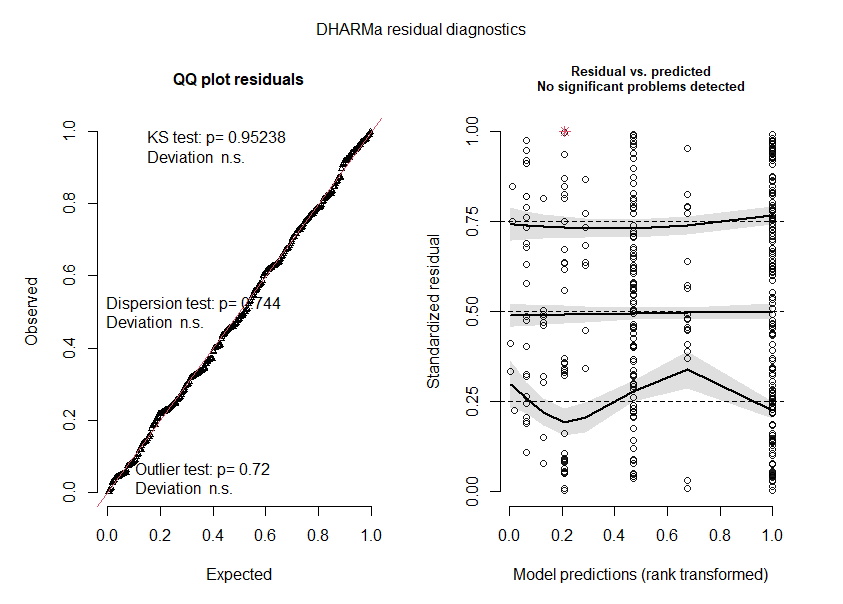


**3. References**

Barton, K. 2020. MuMIn: Multi-Model Inference. R package version 1.43.17. <https://CRAN.R-project.org/package=MuMIn>

Hartig. F. 2021. DHARMa: Residual Diagnostics for Hierarchical (Multi-Level / Mixed) Regression Models. R package version 0.4.3. <https://CRAN.R-project.org/package=DHARMa>

Mazerolle, Marc M. 2020 AICcmodavg: Model selection and multimodel inference based on (Q)AIC(c). R package version 2.3-1. <https://cran.r-project.org/package=AICcmodavg>.
